# Possible Mechanisms of the Prevention of Doxorubicin Toxicity by Cichoric Acid—Antioxidant Nutrient

**DOI:** 10.3390/nu10010044

**Published:** 2018-01-05

**Authors:** Agata Jabłońska-Trypuć, Rafał Krętowski, Monika Kalinowska, Grzegorz Świderski, Marzanna Cechowska-Pasko, Włodzimierz Lewandowski

**Affiliations:** 1 Department of Chemistry, Biology and Biotechnology, Faculty of Civil Engineering and Environmental Engineering, Białystok University of Technology, Wiejska 45E Street, 15-351 Białystok, Poland; m.kalinowska@pb.edu.pl (M.K.); g.swiderski@pb.edu.pl (G.Ś.); w.lewandowski@pb.edu.pl (W.L.); 2 Department of Pharmaceutical Biochemistry, Medical University of Bialystok, 15-222 Białystok, Poland; r.kretowski@umb.edu.pl (R.K.); mapasko@gmail.com (M.C.-P.)

**Keywords:** cichoric acid, doxorubicin, fibroblasts, skin, oxidative stress

## Abstract

Skin is the largest organ in the human body, and which protects organism against unfavorable external factors e.g., chemicals, environment pollutants, allergens, microorganisms, and it plays a crucial role in maintaining general homeostasis. It is also an important target of oxidative stress due to the activity of oxygen reactive species (ROS), which are constantly generated in the fibroblasts in response to exogenous or endogenous prooxidant agents. An example of such compound with proved prooxidant activity is Doxorubicin (DOX), which is an effective anticancer agent belongs in anthracycline antibiotic group. Increasingly frequent implementation of various strategies to reduce undesirable DOX side effects was observed. Very promising results come from the combination of DOX with dietary antioxidants from the polyphenol group of compounds, such as cichoric acid (CA) in order to lower oxidative stress level. The aim of this work was to evaluate the influence of CA combined with DOX on the oxidative stress parameters in fibroblasts, which constitute the main cells in human skin. We also wanted to examine anti-apoptotic activity of CA in fibroblasts treated with selected concentrations of DOX. Results obtained from the combination of DOX with CA revealed that CA exhibits cytoprotective activity against DOX-induced damage by lowering oxidative stress level and by inhibiting apoptosis. The present finding may indicate that CA may serve as antioxidative and anti-apoptotic agent, active against DOX-induced damage.

## 1. Introduction

Skin is the largest organ in the human body. It protects the organism against unfavorable external factors e.g., chemicals, environment pollutants, allergens, microorganisms, and it plays a crucial role in maintaining general homeostasis [1]. Therefore, it is also an important target of oxidative stress due to the activity of reactive oxygen species (ROS), which are constantly generated in the fibroblasts and keratinocytes in response to exogenous or endogenous prooxidant agents [2]. Furthermore, ROS produced during normal skin metabolism, constitute a part of proper skin functionality and usually cause a little damage due to the activity of intracellular mechanisms, which reduce their harmful properties. It is worth noting that intensive or prolonged oxidative stress may overwhelm antioxidant defense mechanisms of the human skin and contribute to the skin disorders development, e.g.,: skin aging, dermatitis, allergic reactions or even skin neoplasms [3]. Main constituents of skin are fibroblasts, which arise during embryogenesis from embryonic mesenchyme. A certain amount of spindle-shaped mesenchymal cells differentiate into supporting cells—fibroblasts. They are usually elongated, spindle-shaped, with several projections lying in one plane. They have one cell nucleus, round or oval with distinct nucleoli. Sometimes several nucleoli within the nucleus can be observed. Fibroblasts are the main cells that build up the dermis. Skin consists of three layers: epidermis, dermis and subcutaneous tissue. The epidermis forms a surface layer that stays in contact with the external environment and is built principally of keratinocytes that synthesize keratin—a component of the stratum corneum of the epidermis. The skin is composed of two layers: papillary and reticular [4,5].

Free radicals as atoms or compounds, which have at least one unpaired electron in the outer orbit, are mainly produced in the mitochondria during electron flow through the mitochondrial respiratory chain, and they increase their reactivity through oxidation-reduction mechanisms. About 2% of oxygen consumed by the respiratory chain undergoes simultaneous reduction causing the formation of ROS. As a result of these processes, superoxide anion radicals are formed, that initiate a whole cascade of reactive oxygen species, such as hydroxyl radical, hydrogen peroxide and singlet oxygen. ROS are generated in cells in different ways, but their main source are mitochondria, which consume about 90% of the oxygen needed by the cells. Mitochondria are also most often damaged by ROS, and with age they degenerate fairly quickly. Under normal conditions ROS are permanently removed by a complex antioxidant system, but in pathological conditions there is an imbalance between prooxidants and antioxidants. This imbalance can lead to oxidative stress and subsequently overproduction of ROS, which modify cellular components such as DNA, proteins and lipids [6,7]. Oxidative stress largely affects the skin, which occupies the largest area of the human body and is a protective coating for the internal organs. The skin structure allows it to perform physical and biochemical protection functions. Due to its functions and localization in the human body, skin is the most exposed for oxidative stress tissue. It is most susceptible to oxidative damage, which results from the presence of potential biological targets for free radical reactions in its components. 

Air pollution, naturally occurring airborne gases such as ozone or high concentrations of oxygen, ionizing and non-ionizing radiation, bacteria, viruses and various exogenous toxins and chemicals can be considered as exogenous sources of factors that promote oxidative stress in the skin. Oxidative stress can also be caused by physical damage to the skin, such as burns or injuries [8,9]. On the other hand, endogenous sources of oxidative damage are generated by enzymes involved in the autoxidation of endo- and exogenous compounds, as well as by lipoxygenases and cyclooxygenases during eicosanoid metabolism. Nitric oxide synthase is an example of an enzyme that produces NO (nitric oxide) radicals directly in the skin. The source of ROS may also be neutrophils activated by a bacterial invasion in which a respiratory burst is observed.

One of the classes of compounds, which activity in the human organism is based on the generation of oxidative stress, mainly in cancer cells, are anticancer drugs. An example of such compound with proved prooxidant activity is Doxorubicin (DOX). DOX is an effective anticancer agent from anthracycline antibiotic group, which has a large spectrum of activity and it is being used alone or in combination to treat a variety of tumors, both haematological and solid, such as breast cancer [10,11]. It inhibits cell proliferation, induces oxidative stress, inhibits topoisomerase II, and finally leads to the cell death mainly through apoptosis [12,13,14]. DOX tends to accumulate in mitochondria, causing changes in their structure and function. However, the main cause of DOX side effects observed in human is an extremely high level of oxidative stress within the cell, eventually causing apoptotic cell death [15,16]. A generation of free radicals caused by DOX can be described in two ways. The first one is an enzymatic mechanism consisting of the formation of semiquinone free radicals by the activity of NADPH-dependent reductases, which cause a one—electron reduction of DOX to DOX-semiquinone. Redox transformations of DOX-derived quinone/semiquinone provide superoxide radicals in the presence of oxygen [17,18]. The second mechanism has non-enzymatic background and it involves reaction with an iron (Fe^3+^), which produces Fe^2+^-doxorubicin free radical complex. This Fe-DOX complex is able to reduce oxygen to hydrogen peroxide and other free radicals. In our previous experiment regarding DOX influence on breast cancer cells we revealed that DOX-metal complexes are even more efficient than a parent drug [10,17]. 

Increasingly frequent implementation of various strategies to reduce undesirable DOX side effects was observed. Besides dose regulation, combined therapies with antioxidants, especially plant origin substances, are being used. Very promising results come from the combination of DOX with dietary antioxidants from the polyphenol group of compounds in order to lower oxidative stress level [19]. One of the antioxidative agents with promising properties is cichoric acid. Cichoric acid (CA), a dicaffeyltartaric acid, was identified in a variety of edible plants and vegetables, such as: iceberg lettuce, basil, *Cichorium intybus* L., dandelion, *Echinacea purpurea* and *Orthosiphon stamineus*. Many of above-mentioned plants are being used in folk medicine. CA exhibits many biological properties including anti-hyaluronidase activity, protection of collagen from possible free radical damage, antiviral activity, promoting phagocyte activity and free radical scavenger properties [20,21]. According to the literature, CA also inhibits HIV (human immunodeficiency virus) integrase, which is responsible for the HIV DNA copy integration with the DNA of the host cell [22]. It should be also mentioned that CA has the ability to influence an immune response in chronically stressed mice, causing a significant decrease in stress level [23,24]. In addition recent research revealed that CA enhances glucose uptake in muscle cells and subsequently stimulates Langerhans islets to the secretion of the insulin [25]. Despite so many beneficial activities of CA described in literature, no studies exist regarding its antioxidative properties against DOX-induced oxidative stress in normal human fibroblasts.

Several studies have shown that CA reveals its general antioxidative properties, which may counteract doxorubicin-induced cytotoxicity, but they were conducted on cancer cell lines and they have not clarified the mechanisms of its action. Therefore, with our study, we wanted to determine if CA acts as an antioxidant against doxorubicin—induced oxidative stress and if it acts via antioxidative and/or antiapoptotic pathway. We tested it in human normal fibroblasts cell line, because fibroblasts are very responsive to chemical signals and changes made to this signals can affect other cells. Fibroblasts constitute also main cell type of the skin, which is exposed for anticancer drugs side effects. Therefore strategies to attenuate the toxic effects of doxorubicin, such as combined therapy with dietary antioxidants (e.g., CA) are implemented. 

## 2. Materials and Methods

### 2.1. Reagents 

Dulbecco’s modified Eagle’s medium (DMEM), containing glucose at 4.5 mg/mL (25 mM), penicillin, streptomycin, trypsin–EDTA (ethylenediaminetetraacetic acid), FBS (Fetal Bovine Serum) and PBS (Phosphate-buffered saline) (without Ca and Mg) were provided by Gibco (San Diego, CA, USA), GSH/GSSG-Glo™ Assay kit, Caspase-Glo^®^ 3/7 Assay kit, Caspase-Glo^®^ 9 Assay kit were provided by Promega, Madison, WI, USA. MTT reagent was purchased from Sigma-Aldrich (St. Louis, MO, USA). Acridine orange and ethidium bromide were obtained from Sigma-Aldrich (St. Louis, MO, USA). Dichlorodihydrofluorescein diacetate assay (DCFH-DA) was provided by Sigma-Aldrich, St. Louis, MO, USA. SDS (Sodium dodecyl sulfate), TCA (Trichloroacetic acid), TBA (Thiobarbituric acid), Folin-Ciocalteu reagent were provided by Sigma-Aldrich and DTNB (Ellman’s Reagent) (5,5-dithio-bis-(2-nitrobenzoic acid)) by Serva. Cichoric acid, chlorogenic acid, caffeic acid, quercetin, DPPH (2,2-diphenyl-1-picrylhydrazyl), trolox, H_2_O_2_, horseradish peroxidase, phosphate buffer pH = 7 were purchased from Sigma-Aldrich Co. and used without purification. Methanol was purchased from Merck (Darmstadt, Germany).

### 2.2. Anti-/Pro-Oxidant Activity

Antiradical activity was determined according to the DPPH assay described by [26]. The methanolic solutions of cichoric, chlorogenic and caffeic acids, quercetin and DPPH were prepared just before analysis. 1 mL of tested compound and 2 mL of DPPH was added to each tube, vortexed and incubated in the darkness for 1 h at 23 °C. The final concentrations of phenolic compounds were 8, 5, 1 and 0.5 μM, and DPPH was 40 μM. The absorbance of solutions was measured at 516 nm against methanol as the blank using Agilent Carry 5000 spectrophotometer (Agilent, Santa Clara, CA, USA). The control sample consisted of 2 mL of DPPH solution and 1 mL of methanol. The antiradical activity of phenolic compounds was calculated as the percent of DPPH inhibition according to the equation:

$$\%I=\frac{A_{\mathrm{control}}^{516}-A_{\mathrm{sample}}^{516}}{A_{\mathrm{control}}^{516}}\times 100\%$$

where: %I—% inhibition of DPPH radical, $A_{\mathrm{conrol}}^{516}$—absorbance of the control, $A_{\mathrm{sample}}^{516}$—absorbance of the sample. 

The pro-oxidant activity of tested compounds was determined as the rate of oxidation of trolox according to the procedure described by Zeraik et al. [27]. 100 μM trolox, 50 μM H_2_O_2_, 0.01 μM horseradish peroxidase in phosphate buffer (pH = 7) and 0.25 μM tested substances were incubated at 25 °C. The measurement was conducted at 272 nm using Agilent Carry 5000 spectrophotometer. 

### 2.3. Cell Culture

The effect of CA, DOX and CA-DOX was examined in normal human skin fibroblast cell line (CRL1474), which was obtained from American Type Culture Collection (ATCC). Cells were maintained in DMEM (Gibco) supplemented with 10% FBS (Gibco), penicillin (100 U/mL), and streptomycin (100 μg/mL) at 37 °C in a humidified atmosphere of 5% CO_2_ in air. The viability of cells was estimated at CA concentrations of: 0.5 µM, 1 µM, 10 µM, 50 µM, 100 µM, 200 µM and 300 µM; DOX concentrations of: 0.09 µM, 0.18 µM, 0.38 µM, 0.75 µM, 1.5 µM, 3 µM and 6 µM; mix of CA and DOX in concentrations: 0.5 µM CA + 3 µM DOX, 1 µM CA + 3 µM DOX, 10 µM CA + 3 µM DOX, 50 µM CA + 3 µM DOX, 100 µM CA + 3 µM DOX, 200 µM CA + 3 µM DOX and 300 µM CA + 3 µM DOX. Caspases 3/7 activity, caspase 9 activity, SH (thiol) group content, TBARS (Thiobarbituric acid reactive substances) content, GSH/GSSG (Glutathione/Oxidized Glutathione) ratio, ROS content and apoptosis were examined at CA concentrations of 300 µM, DOX concentration of 3 µM and the mixture of these two compounds (CA + DOX, concentrations: 300 µM + 3 µM, respectively). Fibroblasts were seeded in 96-well plates at a density 2 × 10^4^ cells/well. The cells were cultured for 24 h and 48 h and were treated with DOX in a concentration of 3 μM, CA in a concentration of 300 μM and with a mixture of DOX and CA (3 μM + 300 μM, respectively). Cells were seeded in: (a) transparent plates for cytotoxicity tests; (b) white plates for determination of caspase 3/7 and 9 activity, determination of GSH/GSSG ratio; (c) black plates for intracellular ROS detection.

### 2.4. Chemical Treatment of Cells

CA was stored in a refrigerator at temperature 4 °C. CA was dissolved in TrisHCl buffer. The compound was added to the cultured cells for a final concentration in the range of 0.5 µM to 300 µM. DOX was stored in a freezer at temperature −20 °C. The compound was added to the culture cells for a final concentration in the range of 0.09 µM to 6 µM. CA and DOX were both added to the cultured cells in selected concentrations. The control cells were incubated without the tested compounds. 

### 2.5. CA, DOX and CA-DOX Cytotoxicity

CA, DOX and CA-DOX cytotoxicity were measured according to the method of Carmichael using 3-(4,5-dimethylthiazol-2-yl)-2,5-diphenyltetrazolium bromide (MTT) [28]. The cells cultured for 24 h and 48 h were treated with: 1st—CA in the concentration range from 0.5 µM to 300 µM; 2nd—DOX in the concentration range from 0.09 µM to 6 µM; 3rd—CA combined with DOX: CA in the concentration range from 0.5 µM to 300 µM combined with DOX in the 3 µM concentration. After 24 h and 48 h cells were washed 3 times with PBS and subsequently incubated with 10 µL of MTT solution (5 mg/mL in PBS) for 2 h at 37 °C in 5% CO_2_ in an incubator. Subsequently, 100 µL of DMSO was added and cells were incubated in the dark for the next 2 h. The absorbance was measured at 570 nm in a microplate reader GloMax^®^-Multi Microplate Multimode Reader (Promega Corporation, Madison, WI, USA). The viability of fibroblast cells was calculated as a percentage of control cells, incubated without tested compounds. All the experiments were done in triplicates. 

### 2.6. Determination of Caspase 3/7 Activity

After intended time of incubation medium was removed and Assay reagents were added to the wells. The activity of caspases 3/7 was measured using luminescent assay based on the substrate which contains the tetrapeptide sequence DEVD in a reagent optimized for caspase activity, luciferase activity and cell lysis. The cell lysis was followed by the caspase cleavage of the substrate and generation of the luminescent signal. The luminescence is proportional to the amount of caspase activity present in the sample. Assay is based on the thermostable luciferase activity, which generates stable, “glow-type” luminescent signal. The luminescence was measured in a microplate reader GloMax^®^-Multi Microplate Multimode Reader. All the experiments were done in triplicates. 

### 2.7. Determination of Caspase 9 Activity

After intended time of incubation medium was removed and Assay reagents were added to the wells. The activity of caspase 9 was measured by using luminescent assay based on a luminogenic caspase-9 substrate in a buffer system provided for caspase activity, luciferase activity and cell lysis. The addition of a special Caspase-Glo^®^ 9 Reagent results in cell lysis, followed by caspase cleavage of the substrate, and generation of a “glow-type” luminescent signal. The signal generated is proportional to the amount of caspase activity present in the sample. The assay is based on thermostable luciferase, which generates the stable “glow-type” luminescent signal. The luminescence was measured in a microplate reader GloMax^®^-Multi Microplate Multimode Reader (Promega Corporation, Madison, WI, USA). All the experiments were done in triplicates. 

### 2.8. Total Protein Content in Cells

Fibroblasts (1 × 10^5^ cells/mL) were incubated in 2 mL of culture medium with tested compounds in tissue culture 6-well plates. The determination of protein concentration was performed spectrophotometrically as per Lowry et al. (1951) as described previously [29]. All the experiments were done in triplicates. 

### 2.9. Determination of SH Groups

SH-groups were measured using the method of Rice-Evans (1991) as described previously [29]. Fibroblasts (1 × 10^5^ cells/mL) were incubated in 2 mL of culture medium with tested compounds in tissue culture 6-well plates. All the experiments were done in triplicates.

### 2.10. Determination of TBA Reactive Species (TBARS) Levels

The method of Rice-Evans (1991) was applied in order to measure the level of membrane lipid-peroxidation products (TBARS), as described previously [29]. Fibroblasts (1 × 10^5^ cells/mL) were incubated in 2 mL of culture medium with test compounds in tissue culture 6-well plates. All the experiments were done in triplicates.

### 2.11. Determination of GSH/GSSG

Total glutathione and GSH/GSSG ratio were each assayed in triplicate via GSH/GSSG-Glo™ kit (Promega, Madison, WI, USA) following manufacturer’s instructions. Prior to the assay growth media were removed and cells washed with PBS. Assay is based on a luminescence measurement and detects and quantifies total glutathione (GSH + GSSG), GSSG and GSH/GSSG ratios in cultured cells. Stable luminescent signals are correlated with either the GSH or GSSG concentration of a sample. In this method GSH-dependent conversion of a GSH probe, Luciferin-NT, to luciferin by a glutathione S-transferase enzyme is coupled to a firefly luciferase reaction. Light from luciferase depends on the amount of luciferin formed, which in turn depends on the amount of GSH present. Thus, the luminescent signal is proportional to the amount of GSH. GSH/GSSG ratios are calculated directly from luminescence measurements.

### 2.12. Intracellular ROS Detection

The intracellular ROS level was measured according to Krętowski R. et al. [30]. After diffusion through the cell membrane, DCFH-DA is deacetylated by cellular esterases to a non-fluorescent compound, which is later oxidized by intracellular ROS into a fluorescent 2′,7′–dichlorofluorescein (DCF). After 24 h, the medium was removed, the cells were stained with 10 μM of DCFH-DA in 200 µL PBS at 37 °C, 5% CO_2_ incubator, for 45 min. Next, the dye was removed and replaced with DOX in a concentration of 3 µM, CA in a concentration of 300 µM and with a mixture of DOX + CA (3 µM + 300 µM, respectively) and incubated for 24 h and 48 h. Then, the DCF fluorescence intensity was measured using the GloMax^®^-Multi Detection System (Promega Corporation, Madison, WI, USA) at the excitation wavelength of 485 nm and the emission wavelength of 535 nm. The intracellular ROS generation in fibroblasts cells was shown as the intensity of fluorescence of the DCF. All the experiments were done in triplicates.

### 2.13. Intracellular ROS Detection by Flow Cytometry

The level of intracellular reactive oxygen species (ROS) was determined using dichlorodihydrofluorescein diacetate (DCFH-DA), (Sigma, St. Louis, MO, USA). After diffusion through the cell membrane, DCFH-DA is deacetylated by cellular esterases to a non-fluorescent compound, which is later oxidized by intracellular ROS into a fluorescent 2′,7′–dichlorofluorescein (DCF). The cells (2.5 × 10^5^) were seeded in 2 mL of growth medium in 6-well plates. After 24 h, the medium was removed, the cells were stained with 10 μM of DCFH-DA in 2 mL PBS at 37 °C, 5% CO_2_ incubator, for 30 min. Next, the dye was removed and replaced with DOX in a concentration of 3 µM, CA in a concentration of 300 µM and with a mixture of DOX and CA (3 µM + 300 µM, respectively) and incubated for 24 h and 48 h. Then, the cells were trypsinized, resuspended in DMEM and then in PBS. The DCF fluorescence intensity was measured according to flow cytometry method, by using FACSCanto II cytometer (Becton Dickinson, Franklin Lakes, NJ, USA). Data were analysed using FACSDiva software (version 6, Becton Dickinson, Franklin Lakes, NJ, USA). The intracellular ROS generation in fibroblasts was depicted as the % of DCF positive cells.

### 2.14. Fluorescent Microscopy Assay

For apoptotic and necrotic cells nuclear morphology evaluation fluorescent dyes, such as ethidium bromide (10 µM) and acridine orange (10 µM) were used. The MCF-7 cells were seeded on cell imaging dishes with coverglass bottom with DOX in a concentration of 3 µM, CA in a concentration of 300 µM and with a mixture of DOX and CA (3 µM + 300 µM, respectively) and without tested compound as a control, for 24 h and 48 h. After incubation the cells were washed twice with PBS and then stained with dyes solution in the dark in room temperature, for 10 min. After incubation, staining mixture was removed and the cells were washed with PBS in order to analyze under fluorescent microscope (200× magnification). Ethidium bromide stains only cells that have lost their membrane integrity, while acridine orange stains live and dead cells. The cells were analyzed and photographed via using Leica DM IL fluorescent microscope (Leica Microsystems, Wetzlar, Germany). The following criteria were used: living cells—have regularly distributed green chromatin nucleus, early apoptotic cells characterized by bright green nucleus with condensed or often fragmented chromatin, late apoptotic cells—orange nuclei with chromatin condensation or fragmentation, while necrotic cells showed orange-stained cell nuclei. The cells were counted and percentage of apoptotic cells was the sum of early apoptotic and late apoptotic cells percent.

### 2.15. Statistical Analysis

For parametric data one-way analysis of variance (ANOVA) followed Tukey’s test was applied. Results from five independent experiments were expressed as mean ± standard deviation (SD) of mean for parametric data. Significance was considered when *p* ≤ 0.05. Statistica 13.0 was used.

## 3. Results

### 3.1. Antioxidant (DPPH Assays) and Pro-Oxidant (Trolox Assay) Activity

**Figure 1:**
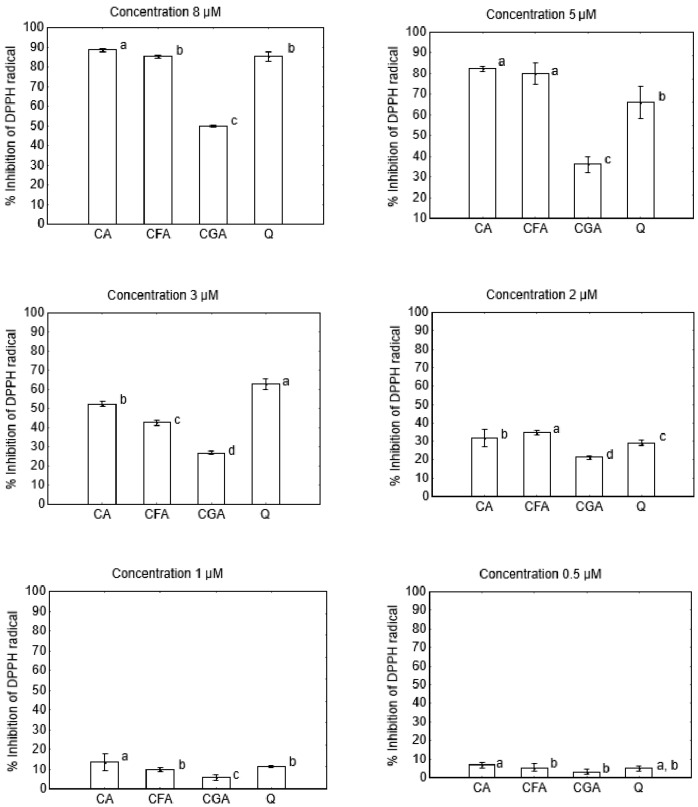
DPPH radical scavenging activity (%) of cichoric acid (CA), caffeic acid (CFA), chlorogenic acid (CGA) and quercetin (Q) for the concentrations 8, 5, 2, 1 and 0.5 μM. The same letter near the means indicate no significant difference (Tukey test, *p* < 0.05).

The anti- and pro-oxidant activity of cichoric acid (CA) was determined and compared with the activity of caffeic acid (CFA), chlorogenic acid (CGA) and quercetin (Q). In Figure 1 the antioxidant properties of selected polyphenolics (determined as antiradical properties against DPPH^•^ radical) are shown. The percent of DPPH^•^ radical inhibition (%I) was the highest for cichoric acid for four out of six studied concentrations and it was equal: 88.44 ± 0.53, 82.37 ± 0.70, 52.52 ± 0.99, 31.81 ± 1.46, 13.60 ± 1.45 and 6.67 ± 0.91%, for the concentrations 8, 5, 3, 2, 1 and 0.5 μM, respectively. Caffeic acid shows slightly lower antioxidant properties than cichoric acid, %I = 85.37 ± 0.41, 80.11 ± 1.67, 42.57 ± 0.48, 9.66 ± 0.76 and 5.45 ± 1.37 for 8, 5, 1 and 0.5 μM, respectively. Quercetin possess similar antioxidant activity as cichoric and caffeic acid, except the 5 and 3 μM concentrations where the %I = 65.96 ± 2.53 and 62.77 ± 0.98, respectively. Chlorogenic acid shows the lowest antioxidant properties among studied compounds, for all studied concentrations (the %I = 49.78 ± 0.14, 36.00 ± 1.98, 26.93 ± 0.38, 21.18 ± 0.59, 5.73 ± 0.63 and 3.01 ± 1.08, respectively; Figure 1). Therefore, taking into account the increasing value of %I as a measure of the antioxidant activity, the studied compounds may be ordered as follows: cichoric acid > caffeic acid~quercetin > chlorogenic acid (for the concentrations 8, 5, 2, 1 and 0.5 μM).

**Figure 2:**
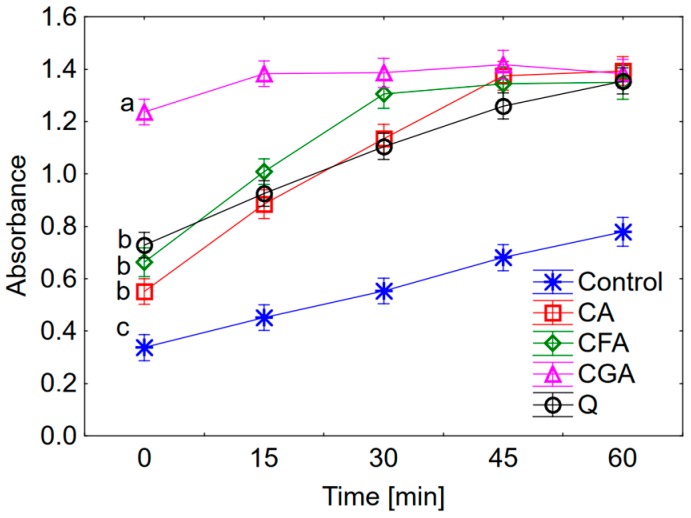
The effect of selected phenolic compounds (0.25 μM) on the oxidation of trolox. CA: cichoric acid, CFA: caffeic acid, CGA: chlorogenic acid, Q: quercetin. The same letters for particular compounds indicate no significant difference (Tukey test, *p* < 0.05).

The pro-oxidant property of phenolic compounds was studied for the concentration 0.25 μM (Figure 2). The rate of oxidation of trolox depends on the type of compounds. For the first 20 min. of measurement chlorogenic acid shows the highest pro-oxidant properties whereas cichoric acid is the lowest (the pro-oxidant activity increases in the series: cichoric acid < quercetin < caffeic acid < chlorogenic acid). For the next 30 min. the pro-oxidant capacity increases as follows: quercetin < cichoric acid < caffeic acid < chlorogenic acid), and after 60 min. all tested compounds reveal similar pro-oxidant properties.

### 3.2. CA, DOX and CA-DOX Cytotoxicity

**Figure 3:**
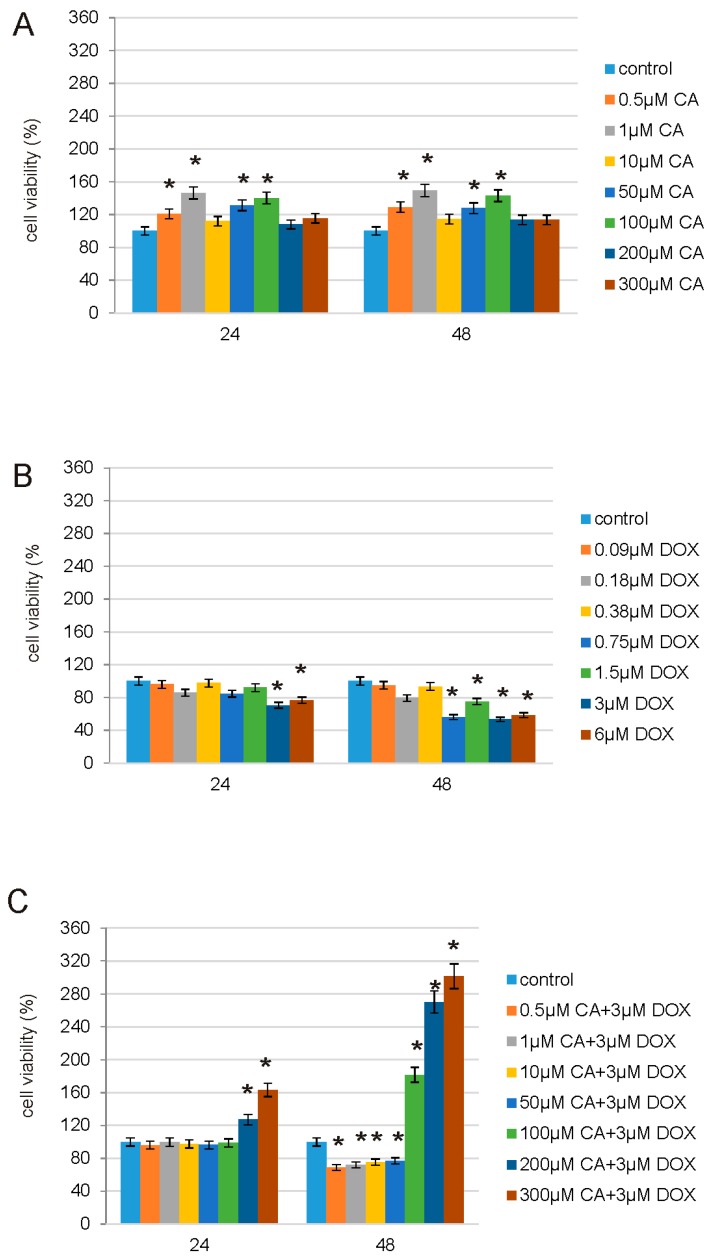
The effect of CA, DOX and CA + DOX on cell viability of fibroblasts. The cells were incubated with 0.5 µM, 1 µM, 10 µM, 50 µM, 100 µM, 200 µM and 300 µM CA for 24 h and 48 h (**A**); 0.09 µM, 0.18 µM, 0.38 µM, 0.75 µM, 1.5 µM, 3 µM and 6 µM DOX for 24 h and 48 h (**B**); mix of CA and DOX in concentrations: 0.5 µM CA + 3 µM DOX, 1 µM CA + 3 µM DOX, 10 µM CA + 3 µM DOX, 50 µM CA + 3 µM DOX, 100 µM CA + 3 µM DOX, 200 µM CA+3 µM DOX and 300 µM CA + 3 µM DOX for 24 h and 48 h (**C**). Mean values from three independent experiments ± SD are shown. Significant alterations are expressed relative to control, untreated cells, and marked with asterisks. Statistical significance was considered if * *p* < 0.05. CA: cichoric acid, DOX: doxorubicin.

Cell viability was determined using MTT assay. CA significantly increased cell proliferation especially after 24 h of exposure (Figure 3A–C). None of the tested CA concentration resulted in a decrease below the control level. The concentration of 1 µM of CA increased the viability of cells by 46% after 24 h and by about 50% after 48 h treatment. Treatment with 100 µM of CA also increased fibroblast cells proliferation by about 40% in both tested incubation times (Figure 3A). DOX treatment caused decrease in fibroblast viability (Figure 3B). The most significant decrease by about 53% was observed under the influence of 3 µM DOX after 48 h treatment. We didn’t observe any increases above the control level in viability of cells under the influence of DOX. Therefore we selected one concentration of DOX, which is 3 µM and combined it with all of the tested concentrations of CA (ranged from 0.5 µM to 300 µM). We observed significant increase in viability of cells after 24 h and 48 h treatment with 200 and 300 µM CA combined with 3 µM DOX (Figure 3C). Simultaneous treatment with 300 µM CA and 3 µM DOX for 48 h increased cell proliferation by about 200% as compared to untreated controls. Based on that experiment we chose one combination of DOX and CA, which was 300 µM of CA and 3 µM of DOX for studying oxidative stress and apoptosis. 

### 3.3. Determination of Caspase 3/7 Activity

**Figure 4:**
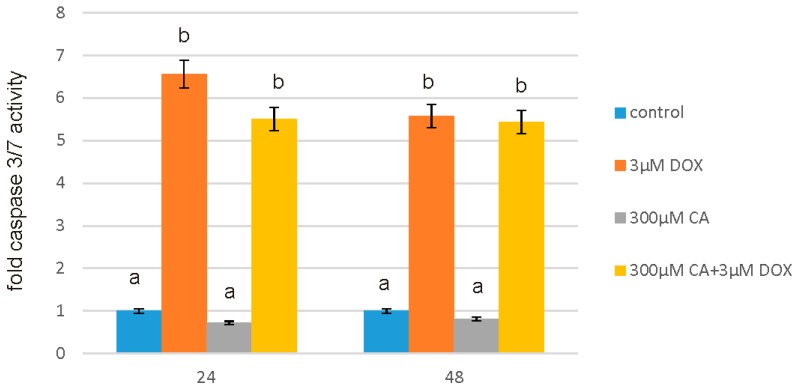
The effect of DOX, CA and CA + DOX on caspase 3/7 activity in fibroblast cells. The cells were incubated with 3 µM DOX, 300 µM CA and DOX (3 µM) combined with CA (300 µM) for 24 h and 48 h Mean values from three independent experiments ± SD are shown. Different letters (a, b) indicate statistical differences (*p* ≤ 0.05) between control, 3 µM DOX, 300 µM CA and DOX combined with CA estimated by Tukey’s test.

Caspase-Glo^®^ 3/7 Assay was used to assess apoptosis level under the influence of the tested substances on fibroblast cell line. The cells were subjected to 3 µM DOX, 300 µM CA and DOX combined with CA in the above-mentioned concentrations for 24 h and 48 h (Figure 4). 300 µM of CA was added to the cell culture 2 h before adding 3 µM DOX. An increased level of apoptosis was observed in the presence of 3 µM DOX and in case of simultaneous treatment with DOX and CA, however when CA was added to the DOX-treated culture the level of apoptosis was decreased as compared to DOX—treated cells. Exposure of fibroblast cells to 300 µM CA significantly decreased caspase 3/7 activity and therefore apoptosis.

### 3.4. Determination of Caspase 9 Activity

**Figure 5:**
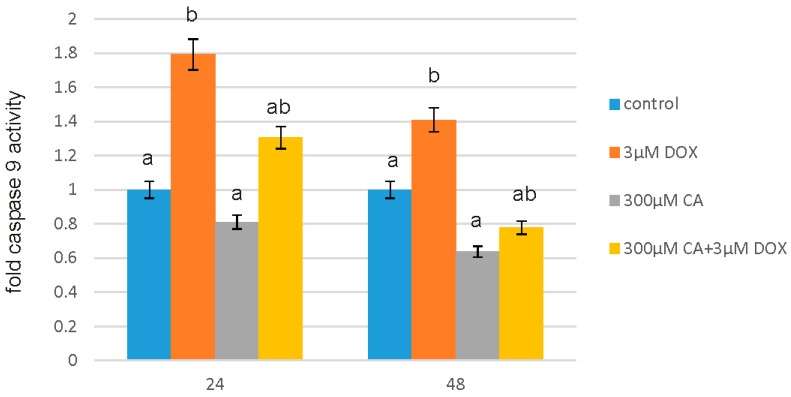
The effect of DOX, CA and CA+DOX on caspase 9 activity in fibroblast cells. The cells were incubated with 3 µM DOX, 300 µM CA and DOX (3 µM) combined with CA (300 µM) for 24 h and 48 h Mean values from three independent experiments ± SD are shown. Different letters (a, b) indicate statistical differences (*p* ≤ 0.05) between control, 3 µM DOX, 300 µM CA and DOX combined with CA estimated by Tukey’s test.

For the measurement of the caspase 9 activity Caspase-Glo^®^ 9 Assay was applied. Similarly as in case of caspase 3/7 activity results, a significant increase in caspase 9 activity was observed under the influence of 3 µM DOX (Figure 5). CA in 300 µM concentration caused a significant decrease in caspase 9 activity. However, the most interesting and significant result is statistically significant decrease in studied caspase activity under the influence of 300 µM CA combined with 3 µM DOX. The level of apoptosis in this case is even lower than in control, untreated cells, which may mean the CA is effective in counteracting the effect of DOX on healthy cell apoptosis.

### 3.5. Determination of SH Groups

**Figure 6:**
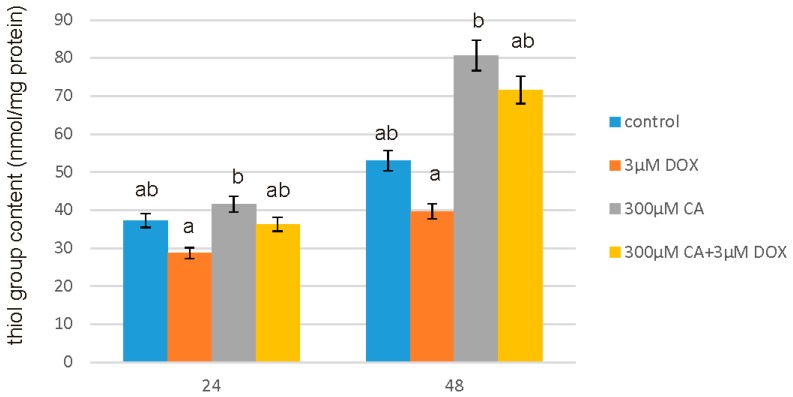
The effect of DOX, CA and CA+DOX on SH group content in fibroblasts. The cells were incubated with 3 µM DOX, 300 µM CA and DOX (3 µM) combined with CA (300 µM) for 24 h and 48 h Mean values from three independent experiments ± SD are shown. Different letters (a, b) indicate statistical differences (*p* ≤ 0.05) between control, 3 µM DOX, 300 µM CA and DOX combined with CA estimated by Tukey’s test.

The effect of DOX and CA on SH group content is shown in Figure 6. To determine the oxidation of the SH group, a spectrophotometric assay with Ellman’s reagent was used. Thiol group content was evaluated as a marker of protein oxidation. A significant decrease in thiol group content of ~25% compared to the control was observed especially after 48 h of treatment at a concentration of 3 µM DOX. Exposure to CA resulted in ~50% increase in the total cellular content of the thiol groups after 48 h of treatment. Obtained results revealed that 300 µM concentration of CA eliminated pro-oxidative effect of DOX on fibroblasts, because we observed a significant increase in thiol group content under the influence of 3 µM DOX in culture pretreated with 300 µM CA. An observed SH group level after 48 h treatment with DOX combined with CA was even higher than it was in control untreated cells. These data indicate that CA is an effective agent in elimination of the oxidative stress, mainly oxidative damage of proteins, caused by DOX. 

### 3.6. Determination of TBA Reactive Species (TBARS) Levels

**Figure 7:**
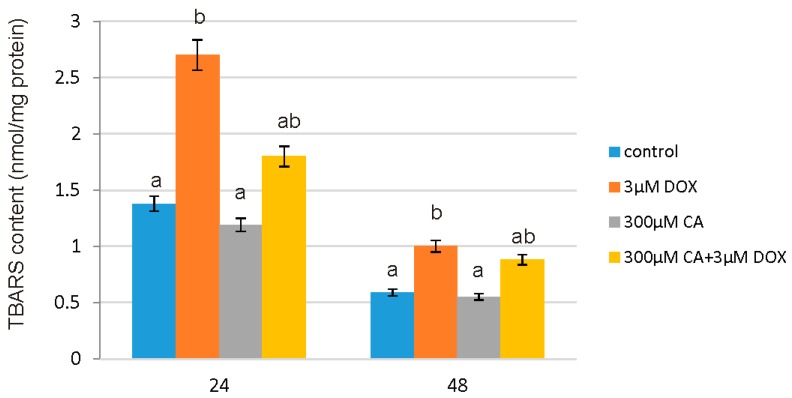
The effect of DOX, CA and CA + DOX on TBARS content in fibroblast cells. The cells were incubated with 3 µM DOX, 300 µM CA and DOX (3 µM) combined with CA (300 µM) for 24 h and 48 h Mean values from three independent experiments ± SD are shown. Different letters (a, b) indicate statistical differences (*p* ≤ 0.05) between control, 3 µM DOX, 300 µM CA and DOX combined with CA estimated by Tukey’s test.

Lipid peroxidation is a process connected with a variety of cellular dysfunctions, which result from the inappropriate modifications of lipid-protein complexes. TBARS content was measured as an index of lipid peroxidation. The results showed significant differences between TBARS levels in the control, DOX, CA and DOX-CA—treated cells (Figure 7). The addition of 3 µM DOX to the cells induced a significant increase in TBARS content of ~95% compared to the control after 24 h treatment. CA at a concentration of 300 µM induced a decrease of ~14% compared to the control observed after 24 h of incubation. CA in 300 µM concentration caused a reduction in TBARS content, which was raised because of DOX influence. The obtained results suggest that CA demonstrates protective properties against TBARS production caused by DOX, and as a consequence it decreases membrane phospholipid peroxidation. 

### 3.7. Determination of GSH/GSSG

**Figure 8:**
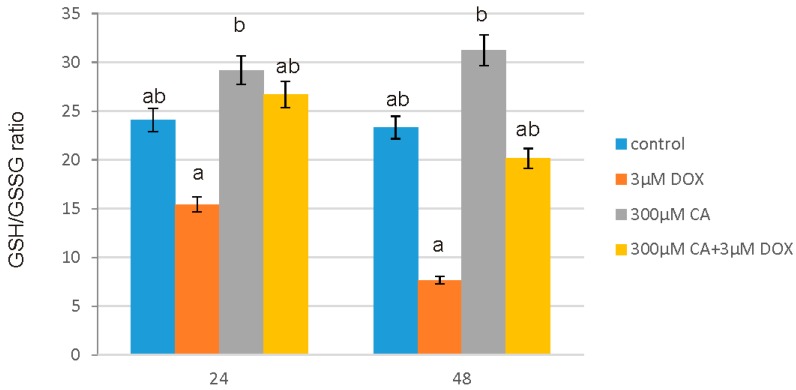
The effect of DOX, CA and CA + DOX on GSH/GSSG ratio in fibroblasts. The cells were incubated with 3 µM DOX, 300 µM CA and DOX (3 µM) combined with CA (300 µM) for 24 h and 48 h Mean values from three independent experiments ± SD are shown. Different letters (a, b) indicate statistical differences (*p* ≤ 0.05) between control, 3 µM DOX, 300 µM CA and DOX combined with CA estimated by Tukey’s test.

Reduced glutathione is one of the most important low mass antioxidants, therefore the estimation of GSH/GSSG ratio constitute an essential study in oxidative stress parameters research. DOX in 3 µM concentration treatment caused a significant decreases in GSH/GSSG ratio after 24 h and 48 h of incubation, while 300 µM of CA treatment resulted in significant increases in both incubation times as compared to the control, untreated cells. Cell culture pre-treatment with CA in 300 µM concentration resulted in an elevated ration of GSH/GSSG after 24 h and 48 h of incubation even after addition of DOX. After 24 h of incubation with the addition of DOX and CA mixture the level of GSH was elevated even as compared to control untreated cells. The effect of DOX, CA and DOX combined with CA on GSH/GSSG ratio is shown in Figure 8. Obtained results revealed an inhibitory influence of DOX and stimulatory effect of CA on GSH amount in fibroblast cell line. 

### 3.8. Intracellular ROS Detection

**Figure 9:**
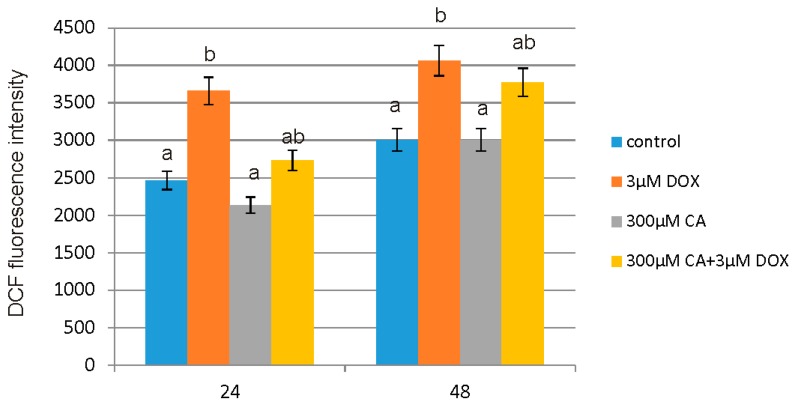
The effect of DOX, CA and CA + DOX on the level of intracellular ROS in fibroblasts. The cells were incubated with 3 µM DOX, 300 µM CA and DOX (3 µM) combined with CA (300 µM) for 24 h and 48 h Mean values from three independent experiments ± SD are shown. Different letters (a, b) indicate statistical differences (*p* ≤ 0.05) between control, 3 µM DOX, 300 µM CA and DOX combined with CA estimated by Tukey’s test.

Figure 9 shows the relative fluorescence intensity of 2′,7′-dichlorofluorescein (DCF) as a percent of control in fibroblast cells incubated with 3 µM of DOX or 300 µM of CA or 3 µM of DOX and 300 µM of CA for 24 and 48 h. An increase in the intracellular ROS production resulted in higher intensity of DCF fluorescence and it was dependent on the concentration of studied compounds. After 24 h incubation of fibroblast cells with 3 µM DOX, the intracellular ROS generation was about 50% higher in comparison to control, untreated cells. The 48 h incubation with DOX resulted in 35% increase in ROS production. CA treatment caused non-significant decrease in intracellular ROS production. Pre-treatment with 300 µM CA significantly reduced ROS content in DOX treated cell culture. These data show inhibitory and protective effect of CA on ROS formation.

**Figure 10:**
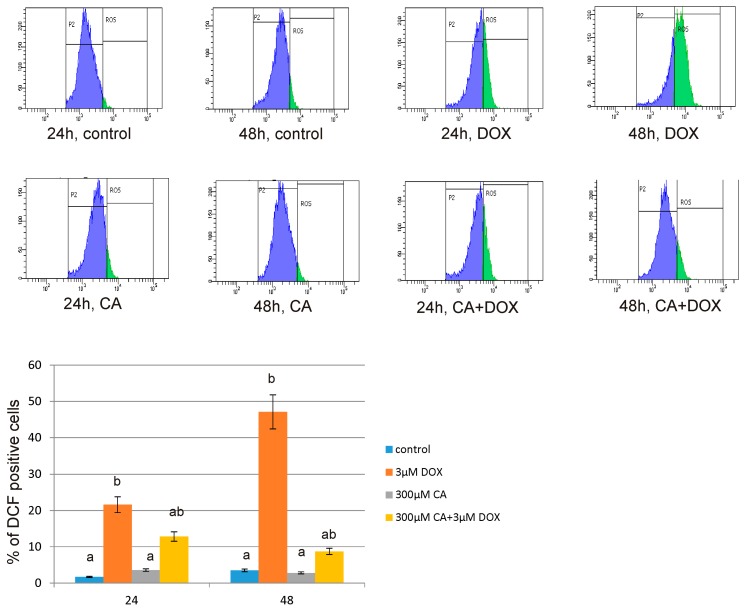
The effect of DOX, CA and CA + DOX on the level of intracellular ROS examined by flow cytometry in fibroblast cells. This Figure shows representative histograms of fibroblasts FACS analysis and the percentage of fibroblast cells generating intracellular ROS. The cells were incubated with 3 µM DOX, 300 µM CA and DOX (3 µM) combined with CA (300 µM) for 24 h and 48 h Mean values from three independent experiments ± SD are shown. Different letters (a, b) indicate statistical differences (*p* ≤ 0.05) between control, 3 µM DOX, 300 µM CA and DOX combined with CA estimated by Tukey’s test

Obtained results were confirmed by using flow cytometry analysis. Figure 10 indicates the effect of DOX, CA and DOX combined with CA on intracellular ROS generation in fibroblasts. It shows the % of DCF positive fibroblast cells incubated with 3 µM of DOX, 300 µM of CA and the mixture of two tested compounds for 24 h and 48 h. Obtained results indicate that DOX in 3 µM concentration is efficient in enhancing oxidative stress level in fibroblasts. The amount of ROS under the influence of DOX was significantly elevated by about 20% after 24 h of incubation and by about 45% after 48 h of incubation as compared to control, non-treated cells. Presented results are statistically significant. Pretreatment with 300 µM of CA caused a significant reduction in ROS content in DOX-treated culture. The difference in ROS formation in cultures treated with CA mixed with DOX and cultures treated with DOX only amounted approximately 40% after 48 h of incubation. This results confirmed protective properties of CA against ROS generation in DOX-treated fibroblasts. 

### 3.9. Fluorescent Microscopy Assay

**Figure 11:**
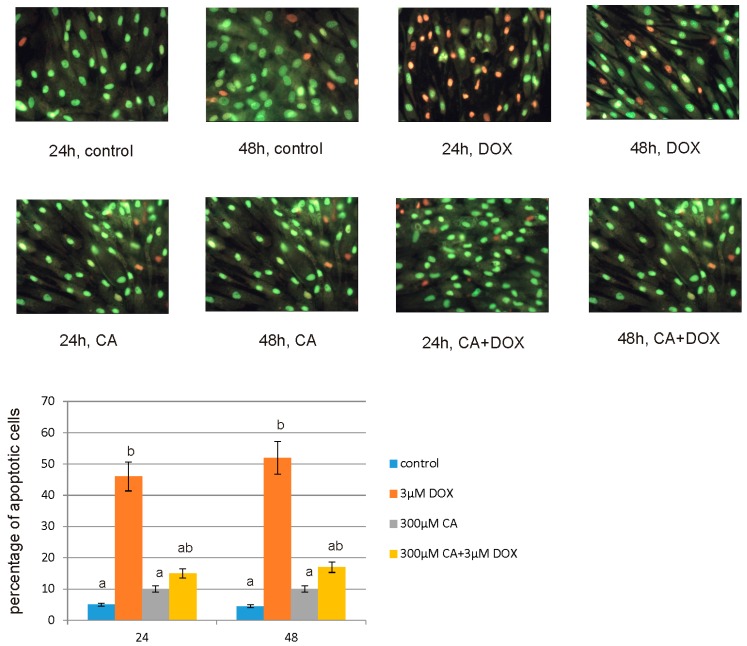
The effect of DOX, CA and CA + DOX on apoptosis and necrosis in the fibroblasts evaluated by fluorescence microscope assay. The cells were incubated in 3 µM DOX, 300 µM CA and DOX (3 µM) combined with CA (300 µM) for 24, and 48 h and stained with acridine orange and ethidium bromide. The cells were photographed under a fluorescence microscope at 200× magnification. We presented representative images from one of three independent experiments and the percentage of apoptotic cells on the graph. Different letters (a, b) indicate statistical differences (*p* ≤ 0.05) between control, 3 µM DOX, 300 µM CA and DOX combined with CA estimated by Tukey’s test

In order to evaluate the apoptotic and necrotic cells morphology and for the confirmation of the apoptosis process measured in above described methods, fluorescent staining was used (Figure 11). Similar to luminescence analysis we observed changes between the control, DOX and CA—treated cells. In control cells lack of chromatin condensation and a few to over a dozen of bright green stained nucleoli were observed. At a 3 µM concentration of DOX, numerous of apoptotic cells with visible chromatin condensation and apoptotic bodies especially after 48 h treatment were seen. DOX treatment also caused chromatin marginalization. However, CA treated cells did not reveal any significant apoptotic changes and CA pretreatment in cells before addition of DOX caused a significant reduction in apoptosis level. 

## 4. Discussion

The selected natural phenolic compounds are common components of human diet and their antioxidant and as well as anti-inflammatory and anticancer properties are widely described, e.g., CA could be beneficial for preventing dermal injury in the setting of DOX treatment [31,32,33,34]. Cichoric acid is a compound with many potentially beneficial properties. It can be obtained from isolated and purified plant and vegetables and it has been described as a one daily nutraceutical which enhances antioxidant activity [35]. The consumption of CA, its derivatives and CA containing plants may significantly influence redox balance in human organism and provide excellent health benefits. CA has been reported as potential anti-diabetic and anti-obesity compound [36]. However, what is important in the context of nutrition in pathological conditions, its oral intake alleviates severe side effects of an excessive alcohol consumption, which was studied in case of acute alcohol-induced hepatic steatosis in mice. Mechanism of CA action in above described study resulted from oxidative stress inhibition and CA anti-inflammation properties [37]. 

The anti- and pro-oxidant activity of cichoric acid was determined and compared with the activity of caffeic acid, chlorogenic acid and quercetin. Cichoric acid is the derivative of caffeic acid and tartaric acid. The presence of the two caffeic acid moieties in the cichoric acid molecule determines its high antioxidant properties. Therefore the caffeic acid was chosen in this comparative study. Chlorogenic acid is another chosen phenolic compound which is an ester of caffeic and quinic acids. It was selected in this study in order to estimate whether the number of caffeic acid moieties or the present of additional structure (e.g., quinic acid fragment) affect the anti-/pro-oxidant properties of molecules. Quercetin was chosen as one of the most intensively studied antioxidants. Obtained results indicate that according to the increasing antioxidant activity the studied compounds may be ordered as follows: cichoric acid > caffeic acid~quercetin > chlorogenic acid. The selected compounds were studied for their pro-oxidative effect on trolox oxidation. In this assay the radicals of polyphenolics are produced in their reaction with H_2_O_2_ catalysed by horseradish peroxidase. Then the phenoxyl radicals react with trolox and cause its oxidation to trolox radicals and trolox quinones. In the same time the phenoxyl radicals are transformed to phenolic compounds. The maximum absorption for trolox quinone is 272 nm. This is a general procedure for study the pro-oxidant activity of phenolic compounds [27]. Obtained results showed that the pro-oxidant activity increases in the series: cichoric acid < quercetin < caffeic acid < chlorogenic acid. Cichoric acid was selected for this study because of its high antioxidant properties and relatively lower pro-oxidant activity compared to compounds which display structural similarities.

An increasing interest in the evaluation of clinical effectiveness of natural extracts, dietary supplements, plant origin antioxidants, especially from the polyphenol group of compounds is being observed. The understanding of the molecular mechanisms underlying dietary antioxidants action in diseases prevention and treatment is important to effectively complete these improvements with healthy lifestyle changes. The aim of this actions is mainly to improve human health, prevent diseases and eliminate some severe side effects of the commonly used chemotherapeutics, especially in the cancer treatment. An example of such chemotherapeutic, widely used in the treatment of a variety of cancers, both hematological malignancies and solid tumors is DOX. However, the clinical application of DOX is limited, which results from its high, cumulative dose-dependent toxicity, especially cardiotoxicity, leading to cardiomyopathy. An observed DOX-induced toxicity may be a result of DOX-induced mitochondrial dysfunction and accumulation of oxidative stress products, such as lipid peroxidation products and/or protein oxidation products [17,38]. 

In order to study possible protective properties of CA on DOX-treated normal human fibroblasts we decided to choose, based on prior experiments, a range of DOX [39] and CA [40] concentrations and estimate the most effective concentrations for both tested compounds. MTT assay revealed that in normal conditions, without any stress factors, CA is efficient in the stimulation of fibroblasts proliferation especially in 0.5 µM, 1 µM, 50 µM and 100 µM concentration. On the other hand, DOX inhibits fibroblasts viability most effectively at the concentration of 3 µM. Therefore, this DOX concentration was chosen for further analysis. The third part of MTT assay showed that pretreatment of fibroblasts with CA (0.5–300 µM) for 2 h before stimulate with DOX was the most effective at 300 µM of CA. Because of that reason, this combination of DOX with CA was applied for the evaluation of the anti-apoptotic and antioxidative effect. Literature data indicate the fact that CA has positive influence on normal cells, e.g., human dermal fibroblasts, where it induces Hepatocyte Growth Factor production on hepatocytes and where it may act as an anti-viral agent [41,42]. 

Reactive oxygen species (ROS) occurring due to too high level of oxidative stress are chemically reactive molecules that may damage main cellular macromolecules, such as DNA, lipids and proteins, subsequently causing genetic mutations. An excessive exposure to ROS may be seriously deleterious because of oxidative damage to important molecules in cell [43]. To assess the effect of CA on proteins in this work we used one of the most widely studied markers of protein oxidation—protein thiol groups content. We observed the oxidative action of DOX, which conforms that DOX exhibits pro-oxidant properties against proteins and this action is connected with its cytotoxic activity. On the other hand stimulation with CA caused a significant increase in thiol group content, which means that tested compound act as an antioxidant. Especially after 48 h of treatment we can observe that prooxidative activity of DOX was effectively reversed by CA treatment. Study by Tsai KL et al. confirmed that pretreatment with CA may inhibit oxidative stress caused by external factors [44]. 

Many natural compounds from the polyphenol group, which also include CA, are characterized by high antioxidant capacities and have been considered as a potential protectors against DOX-induced toxicity. This protective effect may be also connected with the reduction of lipid peroxidation process [19]. In support of this report, we observed that CA does not cause oxidative alterations of membrane lipids and protects against DOX-induced TBARS production, which is probably related with antioxidant properties of CA. Already after 24 h we noticed significant increase in TBARS content in DOX-treated cells and a decrease in cells pretreated with 300 µM of CA. CA activity as a compound that prevents lipid peroxidation process was confirmed by several studies [45,46,47]. Polyphenols exhibit antioxidative properties at several levels, among others they prevent the lipid peroxidation of biological membranes and inhibit the oxidation of lipoproteins and polyunsaturated fatty acids, which is in consistence with our results [48].

Low ROS concentration within the cell is essential for keeping redox balance and for the stimulation of cell proliferation rate. However, an excessive level of accumulated in cell ROS may cause serious damage in protein, lipid and DNA structures through their oxidation and subsequently apoptosis and cell death [49,50]. According to the literature CA, which belongs to the polyphenols group of compounds, may provoke ROS generation in cancer cells and also in preadipocytes, but in normal cells CA was a potent ROS scavenger, reducing ROS accumulation under basal as well as oxidative stress conditions [51]. It is in accordance with our results regarding ROS generation in fibroblast cells. We observed a significant increase in ROS production under the influence of DOX and a decrease in ROS content caused by CA. Pretreatment of fibroblast cells with CA (300 μM) for 2 h before stimulate with DOX significantly inhibited the formation of ROS. Flow cytometry results confirm that intervention with CA mitigated DOX-caused oxidative stress and reduced ROS formation in fibroblasts stimulated with DOX. Therefore we presume that the primary mechanism through which CA alleviates DOX-facilitated cell death of fibroblast cells is its antioxidant function. An observed, DOX-induced significant increase in ROS content was accompanied by a decrease in GSH/GSSG ratio, especially after 48 h of treatment. Similarly as in other tested oxidative stress parameters, GSH/GSSG ratio was significantly reduced after exposure to DOX and these results were effectively reversed by CA treatment. Our results are in accordance with literature data indicating positive influence of selected polyphenols on GSH content. One of the main phenolic compounds of curry spice turmeric is curcumin, the consumption of which brings beneficial effects. It was shown that curcumin plays a protective role against adriamycin-induced toxicity through three main mechanisms: first—it inhibits lipid peroxidation, second – it stabilizes cell membranes and third—it raises glutathione level [52]. Therefore we presume that CA likewise influences GSH level in fibroblast cells. According to Fauser JK et al. a reduced level of the main GSH antioxidant may result in apoptosis and it also may be connected with a reduction of G0/G1 and arrest of the S and M cell cycle phases, together with increased ROS generation [53]. Apoptosis is a programmed cell death that is extremely important for human organism homeostasis maintaining, for tissue development and malignancies treatment. Regulation of this process is main target for anticancer drugs, however chemotherapeutics, such as anthracycline antibiotics, e.g., DOX, may also induce apoptosis in normal, non-cancerous cells. At least two pathways have been described by which an apoptosis occurs: first is an extrinsic death-receptor dependent apoptosis and the second—intrinsic mitochondrial dependent apoptosis. In both pathways selected caspases activation occurs, which means activation of initiator caspases (e.g., caspase-8 and -9) and effector caspases (e.g., caspase-3, -6, and -7) [54,55]. A variety of evidence support also the role of reactive oxygen species in apoptotic cell death. It has been shown that oxygen free radicals are directly involved in the pathogenesis of apoptosis. Caspase 3/7 assay, caspase 9 assay and fluorescent microscopy assay showed that at 3 µM of DOX concentration, numerous apoptotic cells were seen, while pretreatment with 300 µM of CA reduced the number of positive fluorescent cells and decreased the activity of studied caspases significantly. We observed an increase in caspase 3, 7 and 9 activity under the influence of DOX as compared to control non-treated cells, which was accompanied by a decrease in GSH/GSSG ratio and a significant increase in ROS content. Apoptosis is particularly related to the cascade of caspases. Literature data indicated that the release of cytochrome c from the mitochondria into the cytosol, may result in the caspase-9 activation, which subsequently activates effector caspase, such as caspase-3, which causes DNA fragmentation and eventually—cell death [56]. In our study, we found that CA significantly prevented DOX-induced activation of caspase 3 and 7, which is in accordance with literature data that confirms such CA activity. It may thus be hypothesized that this anti-apoptotic effect of CA might be attributed to observed increase in the levels of antioxidant glutathione (reduced GSH) and subsequently to a decrease in oxidative stress level. Tsai KL et al. revealed that CA precluded the activation of caspase 3 and subsequent DNA strand breaks, through the inhibition of the translocation of NF-κB from the cytosol to the nucleus, suppression of Bax and promotion the Bcl-2 expression [44]. In our experiment pretreatment with CA was associated with the inhibition of the downstream apoptotic signaling pathways, finally preventing activation of caspase-3, caspase-7 and caspase -9 induced by DOX. It is suggested that CA is a natural food-derived compound that inhibits apoptosis [57]. Our findings confirm this hypothesis, because under the influence of CA we showed a significant increase in cells proliferation and an decrease in caspase 3/7 and 9 level, which is accompanied by an increase in GSH content and a decrease in ROS content. Anti-apoptotic effect of CA in DOX-treated fibroblasts was confirmed by fluorescence microscopy assay, which indicated that tested compound was efficient both in time and dose-dependent manner. 

## 5. Conclusions 

In conclusion, our findings suggest that CA mitigates DOX-induced oxidative stress and inhibits DOX-facilitated ROS formation. Chicoric acid treatment also inhibited DOX-facilitated apoptosis. It is likely that these beneficial effects contribute to the overall antioxidative and anti-apoptotic function of chicoric acid. However, only in vitro investigations were used to test the cytoprotective effects of chicoric acid from DOX-caused fibroblasts dysfunction. Above results may be the first step in explaining important mechanisms underlying the protective effects of CA observed in DOX cytotoxicity. However, additional studies are indispensable to evaluate long-term effect of CA on dermal injuries caused by DOX treatment. Further analysis must also be conducted on the other skin cell types, such as keratinocytes and melanocytes. Further analysis must also be conducted on the molecular level of the anti-oxidative and anti-apoptotic effects of CA, which may be considered as lead compounds for the development of functional food.
